# Optimization Method for Forecasting Confirmed Cases of COVID-19 in China

**DOI:** 10.3390/jcm9030674

**Published:** 2020-03-02

**Authors:** Mohammed A. A. Al-qaness, Ahmed A. Ewees, Hong Fan, Mohamed Abd El Aziz

**Affiliations:** 1 State Key Laboratory for Information Engineering in Surveying, Mapping and Remote Sensing, Wuhan University, Wuhan 430079, China; alqaness@whu.edu.cn; 2 Department of e-Systems, University of Bisha, Bisha 61922, Saudi Arabia; ewees@du.edu.eg; 3 Department of Computer, Damietta University, Damietta 34517, Egypt; 4 Department of Mathematics, Faculty of Science, Zagazig University, Zagazig 44519, Egypt; abd_el_aziz_m@yahoo.com

**Keywords:** COVID-19, adaptive neuro-fuzzy inference system (ANFIS), forecasting, salp swarm algorithm (SSA), flower pollination algorithm (FPA)

## Abstract

In December 2019, a novel coronavirus, called COVID-19, was discovered in Wuhan, China, and has spread to different cities in China as well as to 24 other countries. The number of confirmed cases is increasing daily and reached 34,598 on 8 February 2020. In the current study, we present a new forecasting model to estimate and forecast the number of confirmed cases of COVID-19 in the upcoming ten days based on the previously confirmed cases recorded in China. The proposed model is an improved adaptive neuro-fuzzy inference system (ANFIS) using an enhanced flower pollination algorithm (FPA) by using the salp swarm algorithm (SSA). In general, SSA is employed to improve FPA to avoid its drawbacks (i.e., getting trapped at the local optima). The main idea of the proposed model, called FPASSA-ANFIS, is to improve the performance of ANFIS by determining the parameters of ANFIS using FPASSA. The FPASSA-ANFIS model is evaluated using the World Health Organization (WHO) official data of the outbreak of the COVID-19 to forecast the confirmed cases of the upcoming ten days. More so, the FPASSA-ANFIS model is compared to several existing models, and it showed better performance in terms of Mean Absolute Percentage Error (MAPE), Root Mean Squared Relative Error (RMSRE), Root Mean Squared Relative Error (RMSRE), coefficient of determination ($R^{2}$), and computing time. Furthermore, we tested the proposed model using two different datasets of weekly influenza confirmed cases in two countries, namely the USA and China. The outcomes also showed good performances.

## 1. Introduction

A large family of viruses, called coronaviruses, are severe pathogens for human beings, which infect respiratory, hepatic, gastrointestinal, and neurologic diseases. They are distributed among humans, birds, livestock, mice, bats, and other wild animals [1,2,3]. The outbreaks of two previous coronaviruses, SARS-CoV and MERS-CoV in 2003 and 2012, respectively, have approved the transmission from animal to animal, and human to human [4]. In December 2019, the World Health Organization (WHO) received notifications from China for many cases of respiratory illness that were linked to some people who had visited a seafood market in Wuhan [5]. Currently, Wuhan city suffers from the spreading of a novel coronavirus, called COVID-19 (previously, it was called 2019-nCoV). In [6], the authors concluded that COVID-19 likely originated in bats, because it is more similar to two bat-derived coronavirus strains. However, the source of the COVID-19 is not confirmed yet, and it requires more investigation. Most of the reported cases are in Wuhan city; therefore, at 10:00 a.m. on 23 January 2020, Wuhan was put on lockdown, in which no people were allowed to go in or out, so, the authorities stopped all public transportation, including airports, trains, metro, and public buses to avoid the spread of COVID-19. Moreover, several cities in Hubei province were put on lockdown. However, many confirmed cases have been recorded in other Chinese cities and about 24 countries globally (up to 3 February 2020) but less than Wuhan city. On 30 January 2020, the Centers for Disease Control and Prevention (CDC) confirmed the human to human transmission of COVID-19. According to the CDC, the virus can be spread by the air, close personal contact, touching surfaces, or objects that contain viral particles, and rarely from fecal contamination. One of the serious problems of the COVID-19 is its incubation period which is up to 14 days as mentioned by [7], and during this period it can spread to others. Moreover, in [8], a Chinese team concludes that the median incubation period is 3 days and it ranged from 0 to 24 days, and the median age is 47.0 years. Therefore, the confirmed cases have increased daily, specifically in China. The spread of such a virus is very dangerous and requires more strict policies and plans, which have already been implemented in many Chinese cities, especially in Hubei province. Thus, it is very critical to forecast the confirmed cases in the upcoming days to enact the necessary protection plans.

Zhao et al. [9] proposed a mathematical model to estimate the real number of COVID-19 cases in the first half of January 2020, which had not been reported. They concluded that the number of unreported cases resulted in 469 cases from 1 to 15 January 2020. In addition, they estimated that after 17 January 2020, the cases had increased 21-fold. Nishiura et al. [10] proposed an estimation model for the infection rate of COVID-19 in Wuhan, China using the data of 565 Japanese citizens who had been evacuated from Wuhan from 29 to 31 January 2020. They conclude that the estimated rate is 9.5%, and the death rate is 0.3% to 0.6%. However, the number of Japanese citizens who had been evacuated from Wuhan is small and insufficient to estimate the infection and death rate. Tang et al. [11] proposed a mathematical model to estimate the transmission risk of COVID-19. They concluded that the basic reproduction number could be 6.47. They also predicted the number of confirmed cases in seven days (23 to 29 January 2020). Moreover, they expected that the peak would be reached after two weeks (from 23 January 2020). In [12], data of 47 patients were used to estimate sustained human-to-human transmission of COVID-19. The author concluded that the transmission is 0.4, and if the time of symptom to hospitalization is half of the tested data, the transmission will be only 0.012. In [13], the authors presented an estimation model for the risk of death from COVID-19. Their estimation results for two different scenarios are 5.1% and 8.4%. They also estimated the reproduction number for both two scenarios as 2.1 and 3.2, respectively. The estimation results showed that the COVID-19 might cause a pandemic.

Previously, many studies were presented for forecasting some epidemics, for example, DeFelice et al. [14] proposed a compartmental model to forecast spillover transmission risk and human West Nile virus (WNV) cases. The proposed model was applied using the historical data of WNY outbreaks of Long Island, New York, for 2001–2014. In [15], a comparison of several time series forecasting models is proposed to forecast hepatitis A virus infection. The authors used 13 years of data of Turkey to test four time series forecasting models, namely multi-layer perceptron (MLP), time-delay neural networks, radial basis function (RBF), and auto-regressive integrated moving average (ARIMA). The comparison outcomes showed that the MLP outperformed other models. In [16], the authors proposed a forecasting model based on ensemble adjustment Kalman filter for seasonal outbreaks of influenza. They evaluated the proposed model using the influenza seasons data of New York City for six years (2003–2008). In addition, Shaman et al. [17] proposed weekly forecasts for seasonal influenza based on susceptible-infected-recovered-susceptible, ensemble adjustment Kalman filter, and influenza-like illness. Moreover, in [18], the authors employed a dynamic model with Bayesian inference to forecast the outbreaks of Ebola in several African countries, namely, Liberia, Sierra Leone, and Guinea. Massad et al. [19] proposed a mathematical model to analyze and forecast the infection of the SARS epidemic. They concluded that the reproduction number for two different communities, Hong Kong and Toronto, were 1.2 and 1.32, respectively. Ong et al. [20] proposed a monitoring and forecasting model for influenza A (H1N1-2009). Furthermore, Nah et al. [21] proposed a probability-based model to predict the spread of the MERS.

The Adaptive Neuro-Fuzzy Inference System (ANFIS) [22] is widely applied in time series prediction and forecasting problems, and it showed good performance in many existing applications. It offers flexibility in determining nonlinearity in the time series data, as well as combining the properties of both artificial neural networks (ANN) and fuzzy logic systems. It has been applied in various forecasting applications, for example, in [23], a stock price forecasting model was proposed using ANFIS and empirical mode decomposition. Chen et al. [24] proposed a TAIEX time series forecasting model based on a hybrid of ANFIS and ordered weighted averaging (OWA). In [25], another time series forecasting method was presented for electricity prices based on ANFIS. Svalina et al. [26] proposed an ANFIS based forecasting model for close price indices for a stock market for five days. Ekici and Aksoy [27] presented an ANFIS based building energy consumption forecasting model. More so, ANFIS is also applied to forecast electricity loads [28]. Kumar et al. [29] proposed an ANFIS based model to forecast return products. Ho and Tsai [30] applied ANFIS to forecast product development performance. However, estimating ANFIS parameters is a challenge that needs to be improved. Therefore, in previous studies, some individual swarm intelligence (SI) methods have been applied to the ANFIS parameters to enhance time series forecasting because these parameters have a significant effect on the performance of ANFIS. The SI methods include the particle swarm optimization (PSO) [31,32], social-spider optimization [33], sine-cosine algorithm (SCA) [34], and multi-verse optimizer (MVO) [35]. For example, in [34] SCA algorithm was applied to improve the ANFIS model to forecast oil consumption in three countries, namely, Canada, Germany, and Japan. In the same context, in [35], The MVO algorithm was used to enhance the ANFIS model to forecast oil consumption in two countries. In addition, in [36] the PSO was used with ANFIS to predict biochar yield. However, individual SI algorithms may stock at local optima. Therefore, one solution is to apply hybrid SI algorithms to avoid this problem. In [37], a hybrid of two SI algorithms, namely GA and SSA, was presented to improve the ANFIS model. The proposed new model called GA-SSA-ANFIS was applied to forecast crude oil prices for long-term time series data. However, the previously mentioned methods suffer from some limitations that can affect the performance of the forecasting output such as slow convergence and the ability to balance between exploration and exploitation phases can influence the quality of the final output. This motivated us to propose an alternative forecasting method dependent on the hybridization concept. This concept avoids the limitations of traditional SI techniques by combining the strengths of different techniques, and this produces new SI techniques that are better than traditional ones.

In the current study, we propose an improved ANFIS model based on a modified flower pollination algorithm (FPA) using the salp swarm algorithm (SSA). The FPA is an optimization algorithm proposed by Yang [38], which was inspired by the flow pollination process of the flowering plants. The FPA was employed in various optimization applications, for example to estimate solar PV parameter [39,40], solving sudoku puzzles [41], feature selection [42], antenna design [43], and other applications [44,45,46,47]. Moreover, SSA is also an optimization algorithm proposed by Mirjalili et al. [48] inspired by the behavior of salp chains. In recent years, the SSA was utilized to solve different optimization problems, such as feature selection [49,50], data classification [51], image segmentation [52], and others [53,54].

The proposed method called FPASSA is a hybrid of FPA and SSA, in which the SSA is applied as a local search method for FPA. The proposed FPASSA starts by receiving the historical COVID-19 dataset. Then a set of solutions is generated where each of them represents the value for the parameters of the ANFIS model. Then the quality of each solution is calculated using the fitness value, and the solution that has the best fitness value is chosen to represent the best solution. Then the probability of each solution is computed. Then the current solution will be updated, either using global or local strategy in FPA. However, in the case of local strategy, the operators of SSA or FPA will be used according to the probability of the fitness value for each solution. The process of updating the solutions is repeated until reaching the stop condition, and the best parameter configurations are used to forecast the number of confirmed cases of COVID-19.

The main contribution points of the current study are as follows:

1. We propose an efficient forecasting model to forecast the confirmed cases of the COVID-19 in China for the upcoming ten days based on previously confirmed cases.
2. An improved ANFIS model is proposed using a modified FPA algorithm, using SSA.
3. We compare the proposed model with the original ANFIS and existing modified ANFIS models, such as PSO, GA, ABC, and FPA.

The rest of this study is organized as follows. The preliminaries of ANFIS, FPA, and SSA are described in Section 2. Section 3 presents the proposed FPASSA, and Section 4 presents the experimental setup and results. We conclude this study in Section 5.

## 2. Material and Methods

### 2.1. Adaptive Neuro-Fuzzy Inference System (ANFIS)

**Figure 1:**
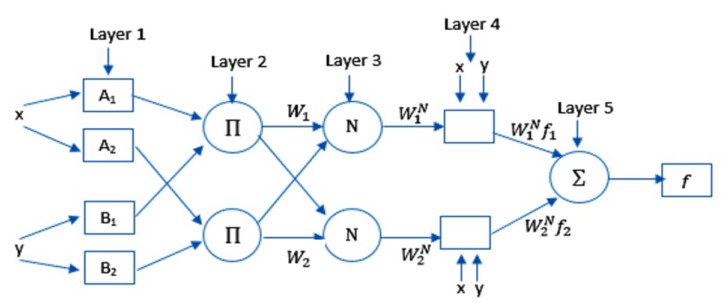
ANFIS model structure.

The principles of the ANFIS are given in this section. The ANFIS model links the fuzzy logic and neural networks [22]. It generates a mapping between the input and output by applying IF-THEN rules (it is also called Takagi–Sugeno inference model). Figure 1 illustrates the ANFIS model where, *y* and *x* define the inputs to Layer 1 whereas, $O_{1i}$ is its output of node *i* that is computed as follows:

(1) $$O_{1i}=\mu_{A_{i}}\left(x\right),\;i=1,2,\;O_{1i}=\mu_{B_{i-2}}\left(y\right),\;i=3,4$$

(2) $$\mu \left(x\right)=e^{-{\left(\frac{x-\rho_{i}}{\alpha_{i}}\right)}^{2}},$$

where μ denotes the generalized Gaussian membership functions. $A_{i}$ and $B_{i}$ define the membership values of μ. $\alpha_{i}$ and $\rho_{i}$ denote the premise parameters set.

The output of Layer 2 (it is also known as the firing strength of a rule) is calculated as follows:

(3) $$O_{2i}=\mu_{A_{i}}\left(x\right)\times \mu_{B_{i-2}}\left(y\right)$$

Meanwhile, the output of Layer 3 (it is also known as the normalized firing strength) is calculated as follows:

(4) $$O_{3i}={\bar{w}}_{i}=\frac{\omega_{i}}{\sum_{\left(i=1\right)}^{2}\omega_{i}},$$

The output of Layer 4 (it is also known as an adaptive node) is calculated as follows:

(5) $$O_{4,i}={\bar{w}}_{i}f_{i}={\bar{w}}_{i}\left(p_{i}x+q_{i}y+r_{i}\right)$$

where $r_{i}$, $q_{i}$, and $p_{i}$ define the consequent parameters of the node *i*. Layer 5 contains only one node; its output is computed as:

(6) $$O_{5}=\sum_{i}{\bar{w}}_{i}f_{i}$$

### 2.2. Flower Pollination Algorithm (FPA)

Flower Pollination Algorithm is an optimization method proposed by Yang [38]. It simulates the transfer of flowers’ pollen by pollinators in nature. This algorithm utilizes the two types of pollination (i.e., self-pollination and cross-pollination). In self-pollination, the pollination occurs with no pollinators, whereas, in cross-pollination, the pollens are moved between different plants. In more detail, the self-pollination can be represented as a local pollination while the cross-pollination can be called global pollination.

The global pollination or cross-pollination can be mathematically formed as follows:

(7) $$x_{i}^{t+1}=x_{i}^{t}+L\left(x_{i}^{t}-F*\right)$$

where $x_{i}^{t}$ defines the pollen *i* at iteration *t*. *L* denotes the pollination’s strength or the step size. F* is the target position or best solution. In some cases, insects can fly with different distance steps for a long space; therefore, Levy fly distribution is applied to simulate this movement.

(8) $$L∼\frac{\lambda \Gamma (\lambda )sin(\pi \lambda /2))}{\pi }\frac{1}{s^{1}+\lambda },\left(s\gg s_{0}>0\right)$$

where λ=1.5. Γ(λ) denotes the gamma function. This distribution is available for large steps s>0.

The self-pollination or local pollination can be mathematically formed as follows:

(9) $$x_{i}^{t+1}=x_{i}^{t}+\epsilon \left(x_{j}^{t}-x_{k}^{t}\right)$$

where $x_{i}^{t}$ and $x_{i}^{k}$ represent pollens from different flower in the same plant. ϵ in the range [0, 1]

The process of pollination can be done using cross-pollination or self-pollination. Therefore, the random variable *p*, in the range [0,1], is used to determine this process.

### 2.3. Salp Swarm Algorithm (SSA)

SSA is an optimization technique introduced by [48]. It simulates the Salps’ behavior in nature. This behavior is called salp chain. The mathematical model of SSA begins by splinting its population into a leader group and followers group. The leader is the front salp, whereas, the followers are the other salps. The search space is determined in *n*-dimensions with *n* variables. Equation (10) works to update the salps’ positions.

(10) $$x_{j}^{1}=\left\{\begin{array}{cc} F_{j}+c_{1}\left(\left(ub_{j}-lb_{j}\right)\times c_{2}+lb_{j}\right) & c_{3}\leq 0\\ F_{j}-c_{1}\left(\left(ub_{j}-lb_{j}\right)\times c_{2}+lb_{j}\right) & c_{3}>0 \end{array}\right.$$

where $x_{j}^{1}$ denotes the leader’s position in *j*-th dimension. $F_{j}$ is the target position. $ub_{j}$ and $lb_{j}$ represent the max and min bounds, respectively. $c_{2}$ and $c_{3}$ denote random numbers in [0,1]. $c_{1}$ is an important parameter; it balances between the exploration and exploitation phases. It is computed as follows:

(11) $$c_{1}=2e^{-{\left(\frac{4t}{t_{max}}\right)}^{2}},$$

where the current loop number is *t* and the max loop’ number is $t_{max}$.

Then, the followers’ position is updated as follows:

(12) $$x_{j}^{i}=\frac{1}{2}\left(x_{j}^{i}+x_{j}^{i-1}\right)$$

where $x_{j}^{i}$ defines the *i*-th position of the follower in *j*-th dimension. *i* > 1.

## 3. The Proposed Method

**Figure 2:**
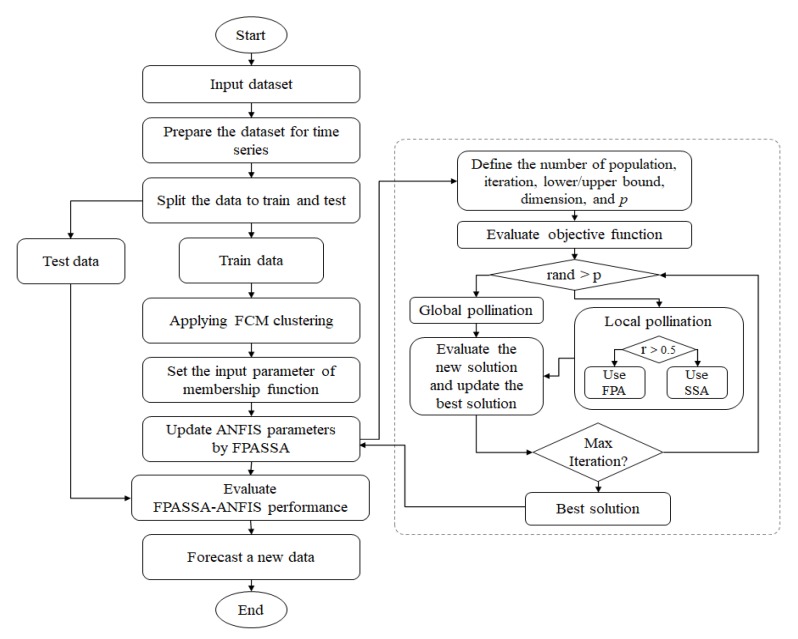
The proposed FPASSA-ANFIS method.

This section explains the proposed FPASSA-ANFIS method. It is a time series method for forecasting the confirmed cases of the COVID-19, as given in Figure 2.

The FPASSA-ANFIS utilizes the improved FPA to train the ANFIS model by optimizing its parameters. The FPASSA-ANFIS contains five layers as the classic ANFIS model. Layer 1 contains the input variables (the historical COVID-19 confirmed cases). Whereas Layer 5 produces the forecasted values. In the learning phase, the FPASSA is used to select the best weights between Layer 4 and Layer 5.

The FPASSA-ANFIS starts by formatting the input data in a time series form. In our case, the autocorrelation function (ACF) was considered. ACF is one of the methods applied to find patterns in the data; it presents information about the correlation between points separated by various time lags. Therefore, in this paper, the variables with ACF greater than 0.2 are considered i.e., 5-lags.

Besides, the training data contains 75% of the dataset, whereas the testing data contains 25% of them. The number of clusters is defined by the fuzzy c-mean (FCM) method to construct the ANFIS model.

The parameters of the ANFIS model are prepared by the FPASSA algorithm. In the training phase, the calculation error (as in Equation (13)) between the real data and the predicted data is used to evaluate the parameters’ quality.

(13) $$MSE=\frac{1}{N_{s}}\sum_{i=1}^{N_{s}}{\left(T_{i}-P_{i}\right)}^{2}$$

where *T* is the real data, and *P* is the predicted data. $N_{s}$ is the sample length. The smaller values of the objective function indicate good ANFIS’s parameter.

On the other hand, the updating phase of the followers’ positions in the SSA algorithm is applied to improve the global pollination phase in the FPA algorithm. In this improvement, there is a random variable (*r*) used to switch between both phases. If r>0.5, then the operators of the SSA is used; otherwise, the operators of the FPA are used. In general, The FPASSA starts by constructing the population (*X*); afterward, the objective function is calculated for each solution. The solution with the lowest error value is saved to the next iteration. This sequence is repeated until meeting the stop condition, which in this paper, is the maximum number of iterations. Then the best solution is passed to train the parameters of the ANFIS model.

After finishing the training phase, the testing phase is started with the best solution to compute the final output. The performance of the proposed method is evaluated by comparing the real data with the predicted data using the performance measures. Finally, the FPASSA produces a foretasted value for confirmed cases of COVID-19 in China in the next day. The steps of the proposed FPASSA are presented in Algorithm 1.
**Algorithm 1** Proposed FPASSA algorithmInput: Historical COVID-19 dataset, size of population *N*, total number of iterations $t_{max}$. Divide the data into training and testing sets. Using Fuzzy c-mean method to determine the number of membership functions. Constructing the ANFIS network. Set the initial value for *N* solutions (*X*). Set t=1. **while**
$t>t_{max}$
**do** Calculate the objective value for each $X_{i}$. **if**
rand>p
**then**  Apply the Global operators of FPA. **else**  **if**
r>0.5
**then**   Using the operators of FPA to update $X_{i}$.  **else**   Using the operators of SSA.  **end if** **end if** **end while** Return the best solution that represents the best configuration for ANFIS. Apply the testing set to the best ANFIS model. Forecasting the COVID-19 for the next ten days.

## 4. Experiment

This section presents the description of the used dataset, the performance measures, the parameter setting for all methods, the experiment results, and discussions.

### 4.1. Datasets Description

The main dataset of this study is COVID-19 dataset. It was collected from the WHO website (https://www.who.int/emergencies/diseases/novel-coronavirus-2019/situation-reports/). It contains the daily confirmed cases in China from 21 January 2020 to 18 February 2020, as shown in Table 1. We used 75% from the dataset to train the model while the rest is used to test it.

Moreover, we evaluated the performance of the proposed method using two datasets of weekly influenza confirmed cases. The first one is called DS1; it was collected from the Centers for Disease Control and Prevention (CDC) (https://www.cdc.gov/flu/weekly/). It starts from week number 40 in 2015 and continues until week number 6 in 2020. Whereas, the second one is called DS2. It was collected from the WHO website (https://www.who.int/influenza). It contains the data of weekly influenza confirmed cases in China from week number 1 in 2016 to week number 8 in 2020.

### 4.2. Performance Measures

The quality of the proposed method is evaluated using a set of performance metrics as follows:

- Root Mean Square Error (RMSE):
  (14) $$RMSE=\sqrt{\frac{1}{N_{s}}\sum_{i=1}^{N_{s}}{\left(YYP_{i}-Y_{i}\right)}^{2}}$$
  where Yp and *Y* are the predicted and original values, respectively.
- Mean Absolute Error (MAE):
  (15) $$MAE=\frac{1}{N_{s}}\sum_{i=1}^{N_{s}}\left|YYP_{i}-Y_{i}\right|$$
- Mean Absolute Percentage Error (MAPE):
  (16) $$MAPE=\frac{1}{N_{s}}\sum_{i=1}^{N_{s}}\left|\frac{YP_{i}-Y_{i}}{YP_{i}}\right|$$
- Root Mean Squared Relative Error (RMSRE):
  (17) $$RMSRE=\sqrt{\frac{1}{N_{s}}\sum_{i=1}^{N_{s}}{\left(\frac{YP_{i}-Y_{i}}{YP_{i}}\right)}^{2}}$$
  $N_{s}$ represents the sample size of the data.
- Coefficient of Determination ($R^{2}$):
  (18) $$R^{2}=1-\frac{\sum_{i=1}^{n}{\left(Y_{i}-YP_{i}\right)}^{2}}{\sum_{i=1}^{n}{\left(Y_{i}-{\bar{Y}}_{i}\right)}^{2}}$$
  where $\bar{Y}$ represents the average of *Y*.

The lowest value of RMSE, MAE, MAPE, and RMSRE refers to the best method. The higher value of $R^{2}$ indicates better correlation for the method.

### 4.3. Parameter Settings

This paper aims to assess the ability of the FPASSA to forecast the COVID-19 by comparing its performance with other methods, namely the ANFIS and the trained ANFIS models using PSO, GA, ABC, FPA, and FPASSA. The parameters’ setting for these models is listed in Table 2.

The common parameters, such as population size, are set to 25 and 100 iterations are applied. Besides, each algorithm is performed for 30 independent runs to fair comparisons. The selected parameters are chosen because they produced good behavior in previous experiments, such as [34,35,55,56].

### 4.4. Performance of FPASSA to Forecast DS1 and DS2

In this section, the performance of the proposed FPASSA to predict the DS1 and DS2 is discussed. It can be concluded from Table 3 that the performance of FPASSA outperformed the compared methods in all measures, whereas the FPA is ranked second. The results of DS2 indicate that the FPASSA is ranked first in terms of RMSE, MAPE, $R^{2}$, and the CPU time. Whereas, the PSO is ranked second, followed by the FPA, GA, then ABC. These results denote that the proposed method can optimize the parameters of the ANFIS model effectively and produce good results in terms of the performance measures.

### 4.5. Influence of FPASSA to Forecast COVID-19

Comparison results between the proposed FPASSA and other models to forecast COVID-19 are given in Table 4. It can be concluded that the FPASSA outperforms other models. For example, by analyzing the results of RMSE, MAE, MAPE, RMSRE, and CPU time(s) it can be observed that the FPASSA achieves the smallest value among the comparison algorithms, and this indicates the high quality of the FPASSA. Meanwhile, the FPA allocates the second rank, which provides better results than the rest of the methods.

**Figure 3:**
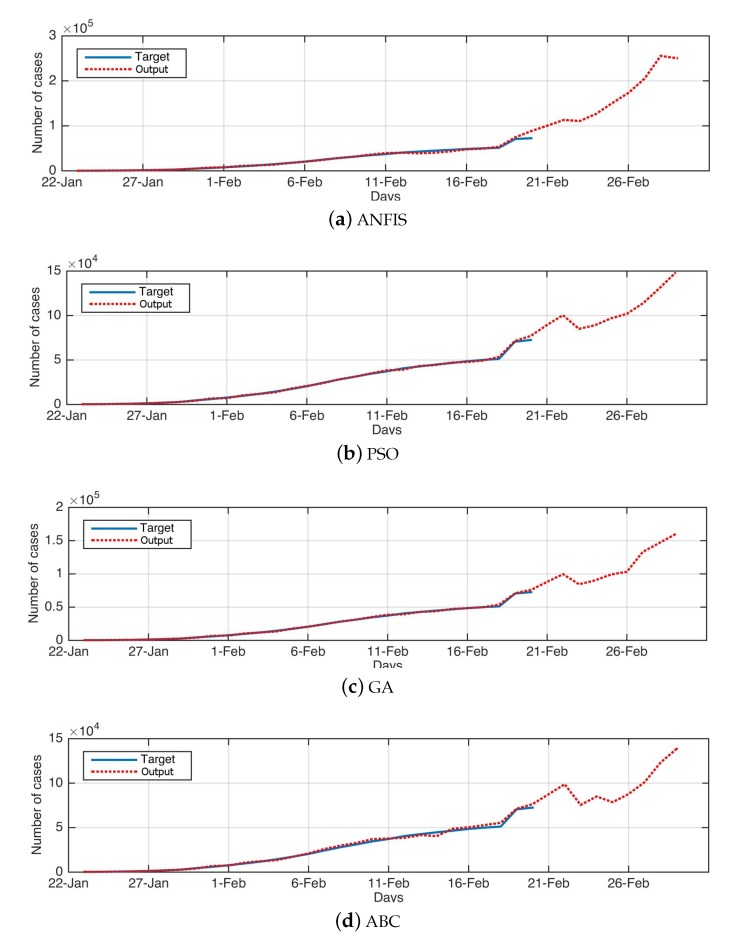

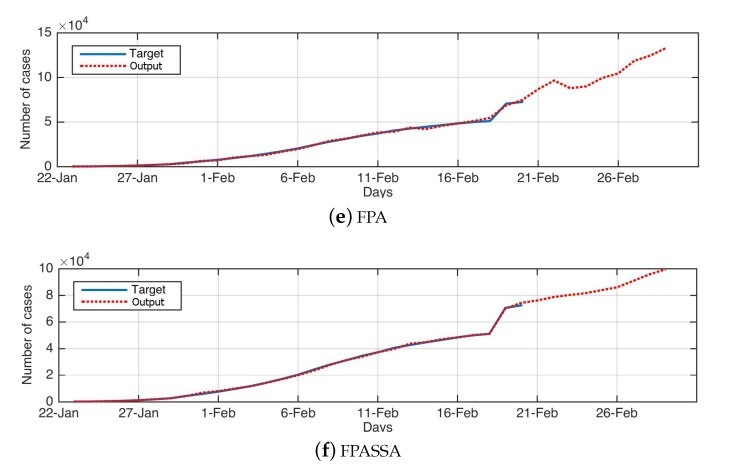
The real data (target) against the forecasted data (output) for all methods.

Moreover, the value of $R^{2}$ refers to the high correlation between the prediction obtained by the proposed FPASSA method and the original COVID-19, which has nearly 0.97. This can also be noticed from Figure 3, which depicts the training of the algorithms using the historical data of the COVID-19 as well as their forecasting values for ten days.

Table 5 depicts the forecasting value for the confirmed cases of the COVID-19 in China from 19/2/2020 to 28/2/2020. From these results, it can be noticed that the outbreak will reach its highest level on the day 28/2/2020. The average percentage of the increase over the forecasted period is 10%, the highest percentage is 12% on 28/2/2020, and the lowest percentage is 8.7% on 19/2/2020.

From the previous results, it can be concluded that the proposed FPASSA-ANFIS has a high ability to forecast the COVID-19 dataset. These results avoid the limitations of traditional ANFIS because of the combination with the modified FPA method. Moreover, the operators of SSA are combined with the local strategy of FPA to enhance their exploitation ability. However, the time computational of the proposed FPASSA method still requires more improvements.

## 5. Conclusions

This paper proposed a modified version for the flower pollination algorithm (FPA) using the salp swarm algorithm (SSA). This modified version, called FPASSA, is applied to improve the performance of the ANFIS through determining the optimal value for its parameters. The developed FPASSA-ANFIS model is applied as a forecasting technique for a novel coronavirus, called COVID-19, that was discovered in Wuhan, China at the end of last year and January of the current year. The proposed FPASSA-ANFIS model has a high ability to predict the number of confirmed cases within ten days. Besides, FPASSA-ANFIS outperforms other forecasting models in terms of RMSE, MAE, MAPE, RMSRE, and $R^{2}$. Furthermore, two datasets of weekly influenza confirmed cases in the USA and China were used to evaluate the proposed method, and the evaluation outcomes showed its good performance. According to the promising results obtained by the proposed FPASSA-ANFIS, it can be applied in different forecasting applications.

## Figures and Tables

**Table 1:** The dataset of the COVID-19.

Date (D/M/Y)	Confirmed	Date (D/M/Y)	Confirmed	Date (D/M/Y)	Confirmed
21/1/2020	278	31/1/2020	9720	10/2/2020	40,554
22/1/2020	309	1/2/2020	11,821	11/2/2020	42,708
23/1/2020	571	2/2/2020	14,411	12/2/2020	44,730
24/1/2020	830	3/2/2020	17,283	13/2/2020	46,550
25/1/2020	1297	4/2/2020	20,471	14/2/2020	48,548
26/1/2020	1985	5/2/2020	24,363	15/2/2020	50,054
27/1/2020	2741	6/2/2020	28,060	16/2/2020	51,174
28/1/2020	4537	7/2/2020	31,211	17/2/2020	70,635
29/1/2020	5997	8/2/2020	34,598	18/2/2020	72,528
30/1/2020	7736	9/2/2020	37,251		

**Table 2:** Parameters’ setting.

Algorithm	Parameters Setting
ANFIS	Max.epochs=100,Errorgoal=0,
	Initialstep=0.01, Decreaserate=0.9,
	Increaserate=1.1
GA-ANFIS	Crossovertype=1,
PSO-ANFIS	wMax=0.9,wMin=0.2,C1=2,C2=2
	cp=1,
	mp=0.01
ABC-ANFIS	a=1,employedbees=N/2,onlookerbees=N/2
FPA-ANFIS	Standardgamma=1.5,Swichprobablity=0.8
FPASSA-ANFIS	$Standard\;gamma=1.5,\;Swich\;probablity=0.8,C_{2}$∈ [0, 1], $C_{3}$ ∈ [0, 1]

**Table 3:** Computational results for datasets of confirmed influenza cases.

Dataset	Method	RMSE	MAE	MAPE	RMSRE	R2	Time
DS1	ANFIS	952	570	37.61	0.551	0.969	-
	PSO	798	494	34.13	0.510	0.978	25.43
	GA	766	480	35.44	0.530	0.98	28.70
	ABC	878	564	39.79	0.593	0.972	49.27
	FPA	618	411	37.69	0.570	0.979	24.58
	**FPASSA**	**609**	**391**	**32.58**	**0.497**	**0.986**	**24.55**
DS2	ANFIS	718	405	64.20	1.198	0.858	-
	PSO	620	**353**	52.07	**0.870**	0.892	31.64
	GA	622	362	87.91	3.216	0.902	34.83
	ABC	696	433	53.30	1.101	0.887	60.87
	FPA	622	371	80.55	3.152	0.898	30.42
	**FPASSA**	**619**	367	**45.02**	0.887	**0.909**	**30.39**

**Table 4:** Computational results for COVID-19.

Method	RMSE	MAE	MAPE	RMSRE	R2	Time
ANN	8750	5413	13.09	0.204	0.8991	-
KNN	12,100	7671	8.32	0.130	0.7710	-
SVR	7822	5354	8.40	0.080	0.8910	-
ANFIS	7375	5523	5.32	0.09	0.9032	-
PSO	6842	4559	5.12	0.08	0.9492	24.18
GA	7194	4963	5.26	0.08	0.9575	27.02
ABC	8327	6066	6.86	0.10	0.7906	46.80
FPA	6059	4379	5.04	0.07	0.9439	23.41
FPASSA	**5779**	**4271**	**4.79**	**0.07**	**0.9645**	**23.30**

**Table 5:** Forecasted confirmed cases of the COVID-19 by the proposed method.

Data	Confirmed Cases (Expected)
19/2/2020	74,406
20/2/2020	76,215
21/2/2020	78,728
22/2/2020	80,332
23/2/2020	81,617
24/2/2020	83,858
25/2/2020	86,115
26/2/2020	90,794
27/2/2020	95,695
28/2/2020	99,453
